# Middle turbinate mucopyocele: a case report

**DOI:** 10.1016/S1808-8694(15)31340-9

**Published:** 2015-10-20

**Authors:** José Antônio Pinto, Pedro Paulo V.C. Cintra, Ana Carla Souza de Marqui, Delmer J.P. Perfeito, Roberto D.P. Ferreira, Rubens H. da Silva

**Affiliations:** ^1^ Director, Nucleus of Otorhinolaryngology and Head and Neck Surgery, Sao Paulo; ^2^ Assistant Physician, Nucleus of Otorhinolaryngology and Head and Neck Surgery, Sao Paulo; ^3^ Resident Physician, Nucleus of Otorhinolaryngology and Head and Neck Surgery, Sao Paulo; Study conducted by the Nucleus of Otorhinolaryngology and Head and Neck Surgery, Sao Paulo, Hospital São Camilo and Hospital Santa Cecília

**Keywords:** mucopyocele, middle turbinate, paranasal sinuses

## Abstract

**M**ucopycocele of the middle turbinate is an uncommon disease. Most mucoceles are situated in the frontal and/or ethmoid sinuses. In this paper we will describe a mucopyocele of the middle turbinate associated with pansinus disease.

## INTRODUCTION

Mucoceles are mucous retention cysts without epithelial lining. Pyocele occurs when mucocele becomes infected^1^. Most mucoceles are located in the frontal and anterior ethmoid sinuses and normally they involve the frontal-ethmoid complex, expanding to the superior-medial region of the orbit, leading to ocular disorders; nasal concha presentation is rare. In the present article, the authors described a case of middle concha mucopyocele associated with chronic pansinusitis, possible disease etiologies and the management adopted for the reported case.

## LITERATURE REVIEW

Even though it is relatively uncommon, mucopyocele are not rare. Most of them occur in the fronto-ethmoidal complex and expand preferably into the orbit, leading to ocular symptoms. However, middle concha mucopyoceles are rare and the etiology is varied and includes previous nasal surgeries, polyp resection and/oronasal tumors and nasal trauma^4^. The first description of middle concha mucopyoceles was made by Badia et al.^1^ in 1994 in a male patient aged 82 years that had been submitted to nasal polyp resection 20 years before, but for 1 year he had started to have nasal obstruction symptoms followed by mucopurulent rhinorrhea on the left with no improvement with clinical treatment (nasal corticoids). Toledano et al.^8^ described a case of middle concha mucopyoceles in a 14-year-old patient that had suffered a nasal trauma as a child and also presented nasal obstruction on the right, followed by mucoid rhinorrhea. Armengot et al.^9^ have also described a case of mucopyoceles in a 65-year-old lady whose main symptom was diplopia with oxophtalmia on the left and medial canthus protuberance on the same eye, but without any nasal symptom. CT scan of the nose and paranasal sinuses showed the presence of soft density content in the left middle concha, expansion against the papyraceous membrane and orbit invasion. These patients underwent surgery to open the infected middle concha, drain the purulent material from inside it and complete resolution of the symptoms. The follow-up of these patients lasted up to 18 months, including nose and paranasal sinuses CT scan, showing complete absence of recurrent or residual disease.

## CASE REPORT

N. C., 64-year-old Caucasian male patient, resident of Sao Paulo. His main complaint was pain on the left brow region. He reported that 30 days before he had started to feel a pain on the left brow region with conjunctiva hyperemia. He went to the ER and was prescribed an eye drop on the left eye for 4 days. He experienced no improvement, so he went back to the ER complaining of left palpebral edema, diplopia, visual acuity impairment and frontal to left headache.

He reported that 40 years before, he had had the same symptoms on the right eye and he was medicated and improved. After that time, he started to present nasal obstruction and intermittent greenish nasal discharge, more on the left, and controlled with clinical approach. He did not report hyaline rhinorrhea, sneezing or nasal pruritus. He reported systemic blood pressure (using captopril, hydrochlortiazide daily). In the ENT examination, we observed palpebral edema and ptosis on the left with present pupil reflex and left conjunctiva hyperemia.

Rhinoscopy: nasal fossa with abundant yellowish rhinorrhea, preventing better visualization of nasal structures; hyperemia and hypertrophy of lower conchae.

**Figure 1:**
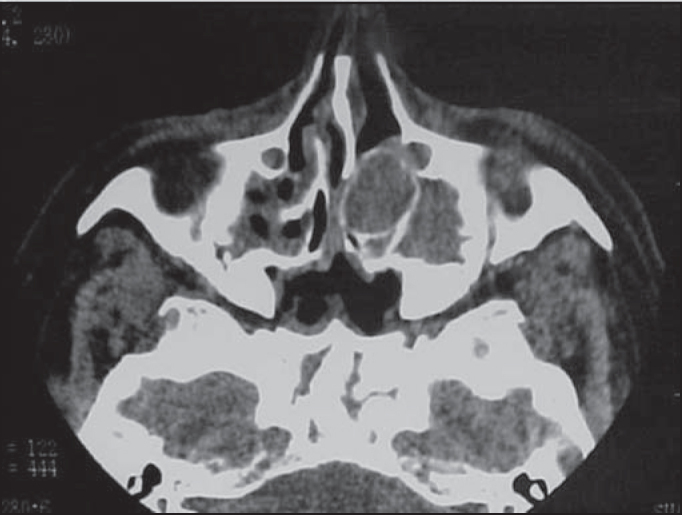
Paranasal sinuses CT scan (axial section) showing pansinusitis and mucopyocele of left middle concha.

Oroscopy: no abnormal findings. Otoscopy: no abnormal findings.We ordered complementary exams: nasal and paranasal sinuses CT scan that revealed left frontal, ethmoidal and maxillary sinuses velamentum with expansion of left middle concha and increase in its density, with no destruction of papyraceous lamina (Figure 1). Considering the diagnosis of mucopyoceles of middle concha and pansinusitis, we indicated endoscopic surgery. The head of middle concha on the left was opened with an incision on its meatal aspect, followed by its resection with moderate drainage of purulent secretion. Next, opening of ethmoidal and frontal sinuses through its natural ostia. In early postoperative care, the patient reported significant improvement of symptoms, and was discharged from hospital with antibiotics and analgesics two days after surgery. Postoperative follow-up (day 7) showed complete improvement of the condition without any nasal symptom.

## DISCUSSION

Mucocele is the preferred term for cysts without epithelial layer caused by trauma. When the term is applied for paranasal sinuses mucoceles, it has a different understanding, because almost all mucoceles in this region are real mucous retention cysts with epithelial layer normally caused by obstruction^2^. Other causes are trauma, nasal polyp, nasal tumor^4^ and/or surgeries. Most mucoceles are located in the frontal and ethmoid sinuses, and in them, anterior cells are the most affected ones.

In the formation of mucoceles there is true bone destruction owing to its expansive character that determines bone reabsorption. In previous studies, Lund et al.^3^ demonstrated that mucocele tissues synthesize more PGE2 (prostaglandin) than normal tissues. The increase in the capacity of inflammatory tissues to synthesize prostaglandin such as PGE2 is normally related with level of inflammatory cytokines (interleukin E, tumor necrosis a factor, vascular adhesion factor), produced as a result of the inflammation. Such cytokines may be responsible for the destruction associated with mucoceles, with consequent reabsorption, rarefaction and local bone expansion, facilitating their widening^3^. This event may be the cause of the bone reabsorption and bone formation areas^8^, observed on the bone walls that surround the mucocele, in which osteoblastic activity is followed by osteogenesis and sclerosis is alternated with areas of active bone destruction^6^. Mucocele, thus, would grow by expanding outwards instead of expanding under the effect of the internal pressure, preserving the sinus mucosa.

Middle concha is a bone plate that is placed inferior-medially to the complex of ethmoid air cells and runs along its anterior limit, and it may be aired (called bullous concha). The first description of bullous concha was made in 1893 by Zuckerlandl^7^. When there is pneumatization of middle concha, there is a large air volume that is enough for its growth, before it may present any symptom. Its pneumatization may occur both anterior and posteriorly, and anteriorly it is potentially prone to the formation of mucopyoceles. The middle concha, in turn, also presents an ostium that connected the air cell lumen with the frontal recess, which may be prone to obstruction caused by local inflammatory edema, or cause obstruction of ethmoidal infundibulum leading to onset of ethmoidal and/or antral disease. Thus, given that it may take a secondary infection to the paranasal sinuses, the bullous middle concha is, therefore, predisposed to the same inflammatory disorders that may occur in any paranasal sinuses, but its prevalence in patients with chronic sinusitis ranges at about 30%^5^.

In the present case, the patient presented chronic rhinosinusitis for many years, and had been clinically treated, but we do not know if appropriately. We then decided for surgery, in which we performed opening of middle concha with drainage of purulent material and resection of meatal aspect, followed by opening of drainage ostium of impaired paranasal sinuses. The patient presented significant improvement at early postoperative care, with complete resolution of the clinical manifestations on postoperative day 7.

**Figure 2:**
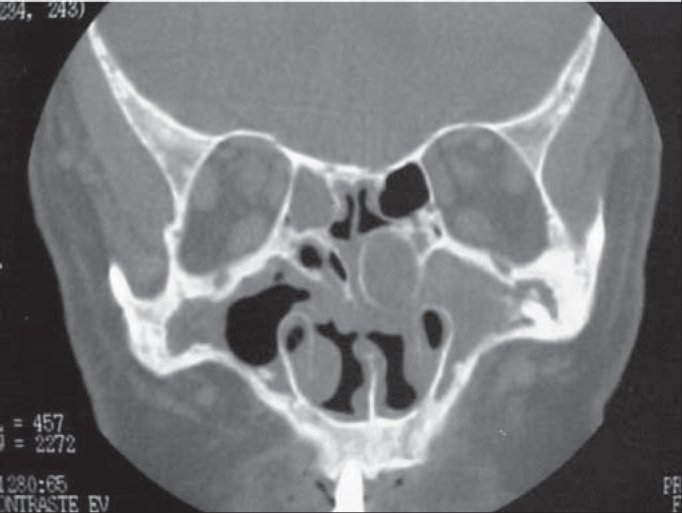
Paranasal sinuses CT scan (coronal section) showing mucopyocele located in the left middle concha.

CT scan and MRI (Magnetic Resonance Imaging) support the diagnosis because they are high resolution exams, allowing differential diagnosis with cysts, expansive masses, erosive lesions, secretion, benign tumors (polyps, papillomas). In this case, paranasal sinuses CT scan allowed the distinction between damage and paranasal sinuses secretion. CT scan showed that apparently it was a nonvascularized lesion, without brain impairment and located on the left middle meatus (Figure 2). Despite the intraoperative confirmation of benign lesion, it is mandatory to perform a histopathological exam to confirm the diagnosis.

## CONCLUSION

Even though middle concha mucopyocele is an infrequent entity, it should be part of the differential diagnosis in cases of rhinosinusopathy with dissatisfactory resolution and in intranasal masses and tumors. Paranasal sinuses CT scans are very important for the diagnosis and in some cases, MRI with gadolinium contrast is also recommended to reach better delineation of the lesion and to distinguish expansive processes of the nose, paranasal sinuses or even cerebral lesions. After confirmation of the diagnosis, endoscopic surgical treatment produces very satisfactory outcomes. In the presence of concomitant paranasal sinuses diseases, such cases should also be surgically treated.
